# Fluorine-free, liquid-repellent surfaces made from ionic liquid-infused nanostructured silicon

**DOI:** 10.1007/s00706-016-1888-2

**Published:** 2016-12-18

**Authors:** Roland W. Bittner, Katharina Bica, Helmuth Hoffmann

**Affiliations:** Institute of Applied Synthetic Chemistry, Vienna University of Technology, Vienna, Austria

**Keywords:** Surface, Monolayers, Nanochemistry, Ionic liquids, Wetting

## Abstract

**Abstract:**

Liquid-repellent surfaces based on slippery liquid-infused porous substrates (SLIPS) were prepared from porous, nanostructured silicon surfaces with different surface functionalization, infused with the polar ionic liquid 1-ethyl-3-methylimidazolium methylsulfate ([C_2_mim]MeSO_4_). Contrary to nonpolar SLIPS based on perfluorinated substrates and infusion liquids, [C_2_mim]MeSO_4_ forms stable SLIPS with high energy surfaces like native silicon (Si–SiO_2_) or ionic liquid-functionalized silicon (Si-[C_3_mim]Cl), whose liquid-repellent properties against low surface tension liquids (toluene, cyclohexane) were demonstrated by very low sliding angles (*α* < 3°) and low contact angle hysteresis (Δ*θ* < 10°). These polar, ionic liquid-based SLIPS present a promising, environmentally benign extension of liquid-infused substrates to natural, high-energy oxide surfaces.

**Graphical abstract:**

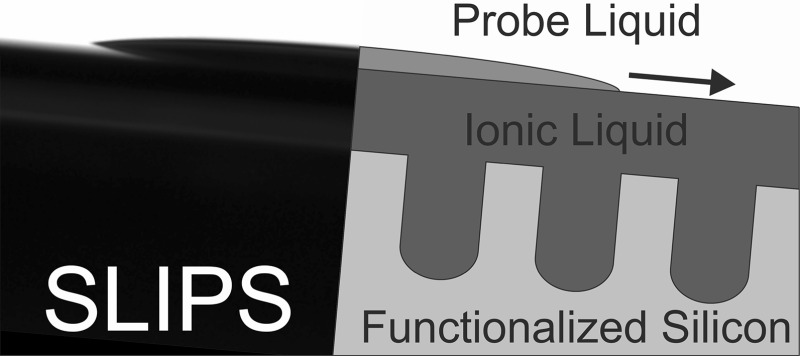

**Electronic supplementary material:**

The online version of this article (doi:10.1007/s00706-016-1888-2) contains supplementary material, which is available to authorized users.

## Introduction

**Fig. 1:**
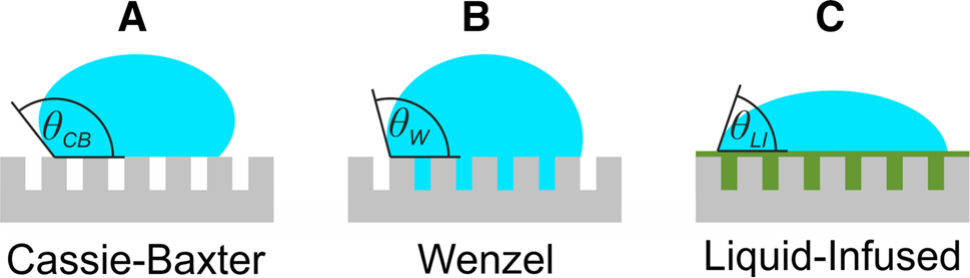
Wetting models for rough, solid surfaces

The wetting properties of a solid surface are commonly characterized by the static contact angle or Young angle *θ*_Y_ [1] of a drop of a probe liquid deposited on the surface and the contact angle hysteresis Δ*θ* [2, 3], which is the difference between the advancing contact angle *θ*_a_ and the receding contact angle *θ*_r_ of a drop rolling down the tilted surface. Liquid repellency is usually expressed by a large Young angle *θ*_Y_ and a small hysteresis Δ*θ*, because a large *θ*_Y_ means a small solid/liquid contact area and a small Δ*θ* means a small surface tilt angle that is required to shed the liquid (the so-called roll-off angle *α*). One of the most challenging tasks in the fabrication of synthetic surfaces with specific wetting properties is to make a surface repellent to all kinds of liquids and natural fluids, in particular inert, nonpolar liquids with low surface tensions [4–7]. Over the past decade, the most common approach to prepare liquid-repellent surfaces has been based on the Cassie–Baxter wetting state [8] (Fig. 1a): a liquid in contact with a rough solid substrate, which is usually coated with an inert, low surface energy substance (most often, a long-chain hydrocarbon or perfluorinated hydrocarbon compound), does not fill the pores of the substrate, but is suspended on the outer, composite surface consisting of a solid fraction *ϕ* and an air fraction (1 − *ϕ*). The resulting contact angle *θ*_CB_ is then a weighted average between the Young contact angle of the plane solid *θ*_Y_ and 180° (contact angle in air). The goal of the Cassie–Baxter approach is to make *θ*_CB_ as large as possible (ideally 180°) and the contact angle hysteresis Δ*θ* as small as possible using substrates with large *θ*_Y_ (low surface energy coatings) and small *ϕ* (e.g., nanostructured or hierarchically structured surfaces) [9]. Thus, a combination of a chemical factor *θ*_Y_ and a structural factor *ϕ* determines the overall wetting and liquid-repellent properties in the Cassie–Baxter case. The main limitation arises from the fact that the CB state is thermodynamically stable only for liquid/solid systems for which the Young angle *θ*_Y_ is larger than a certain critical angle *θ*_C_ = cos^−1^ ((*ϕ* − 1)/(*R* − *ϕ*)), where *R* is the surface roughness (true surface area per unit projected surface area) [10]. This condition can be fulfilled only by a few, selected systems (high surface tension liquids (e.g., water) on low energy surfaces like hydrocarbons or fluoropolymers), whereas for most organic liquids, *θ*_Y_ is always <90° on any plane solid surface and a stable Cassie–Baxter state can never be reached. Instead, the liquid penetrates into the substrate pores and forms the Wenzel wetting state [11] (Fig. 1b), which is characterized by high adhesion between liquid and solid and a large contact angle hysteresis Δ*θ*, i.e., a liquid-attractive instead of a liquid-repellent wetting state. To overcome this limitation and make surfaces repellent even for nonpolar, organic liquids with low surface tension, substantial effort has been put into the development of synthetic substrates with designed surface textures, which prevent the penetration of liquid into the substrate pores and maintain a metastable Cassie–Baxter state [4, 10]. Despite numerous successful examples [12–17], the production of such substrates is generally complex and expensive and practical applications are limited by the instability of this metastable state against external pressure, impact, mechanical damage, high temperatures, etc.

A few years ago, a fundamentally different biomimetic approach for liquid-repellent surfaces was first reported, inspired by the prey-capturing method of the carnivorous *Nepenthes* pitcher plant [18]. This plant captures insects using a slippery, water-lubricated surface at the rim of its pitcher. The rim consists of a porous, irregular microstructure, which fills with water on humid days and remains dry on dry days. Whereas the dry surface is adhesive and non-slippery, the water-filled rim exposes a thin, continuous film of liquid which is held in place by capillary forces of the rim’s microstructure and repels the oil-covered insect feet. Figure 1c shows a model of this liquid-infused wetting state, which is also denoted as slippery liquid-infused porous substrate (SLIPS): the surface of a rough, solid substrate is filled with a lubricating liquid such that the substrate is completely immersed in the liquid. The surface exposed to the ambient phase is no longer a solid, but a thin liquid film of typically a few micrometers thickness. Such a substrate repels liquids, which are immiscible with the lubricating liquid, in a fundamentally different way than a Cassie–Baxter substrate: the solid substrate is no longer in contact with the probe liquid. Its only task is to stabilize the lubricating liquid film through capillary forces and prevent drainage of the film through gravity, shear forces, impact of probe liquid and other external forces. The probe liquid then interacts just with a liquid surface and forms a liquid/liquid interface. Thus, solid/liquid wetting in all Cassie–Baxter based systems is transformed to liquid/liquid wetting in SLIPS-based systems and is governed by completely different rules: The static contact angle and the contact area of a liquid drop on a liquid surface are usually much larger than on a Cassie–Baxter substrate, but the contact angle hysteresis is very small because of the absence of a solid–liquid contact line and related pinning effects, which are the primary cause for contact angle hysteresis [2, 3]. The main requirements for a stable and versatile SLIPS systems are (i) a chemically inert lubricating liquid with low volatility and immiscibility with as many other liquids as possible and (ii) a solid substrate with a suitable microstructure and chemical composition to keep the lubricating film firmly in place. The large majority of practically tested SLIPS consist of fluorinated substrates filled with nonpolar, fluorinated hydrocarbons or polyethers because of their low volatility and low miscibility with other liquids [18–31]. This group of nonpolar SLIPS has been shown to repel a large variety of polar and nonpolar liquids as well as complex fluids like blood, crude oil or vegetable oils [18, 19], can withstand high pressures and temperatures [18, 20] and can even self-heal from minor damage [18, 21]. Successful practical applications include the prevention of biofilm adhesion and biofouling [22–26], self-cleaning surfaces [27, 28], oil–water separation [24], and the fabrication of anti-icing surfaces [29–31]. Yet, some inherent drawbacks of these nonpolar systems are (i) the exclusive use of fluorinated compounds both as substrate coatings and as lubricating liquids, (ii) leaching of the lubricating liquid caused by its low surface tension, and (iii) the fact that most natural surfaces are polar and cannot be used for nonpolar SLIPS without surface pretreatment.

In the present study, we explore the properties of polar SLIPS, consisting of a polar substrate surface with high surface energy and a nonvolatile, polar lubricating liquid. As the substrate material, we have chosen native silicon, which in ambient atmosphere is typically covered by a 1–2 nm-thick oxide layer and therefore represents the ubiquitous group of natural silica-based surfaces. Silica surfaces can also be readily functionalized with a variety of organosilane compounds and allow fine-tuning of their surface energy. Moreover, silicon is also the best-studied material for the fabrication of structured surfaces, and a plethora of methods is available today to make rough, porous substrates to be used as support of the lubricating liquid film in SLIPS. We have chosen for this study a wet-chemical, silver-catalyzed etching technique [32–34], which produces a nanostructured surface consisting of densely packed, uniformly oriented silicon nanowires of about 50–200 nm diameter [34]. In search of environmentally friendlier, fluorine-free and polar lubricating liquids, ionic liquids seemed the most promising candidates, as they combine negligible vapor pressure with tunable physicochemical properties. Of particular importance for the present study is the surface tension, as it determines the wetting properties of the lubricating liquid toward both the substrate and the probe liquid. Surface tensions of ionic liquids range typically between 23 and 60 mN m^−1^ [35, 36] and can be readily adapted for optimized wetting properties of a particular liquid-infused substrate.

## Results and discussion

### Wetting model for liquid-infused substrates

**Fig. 2:**
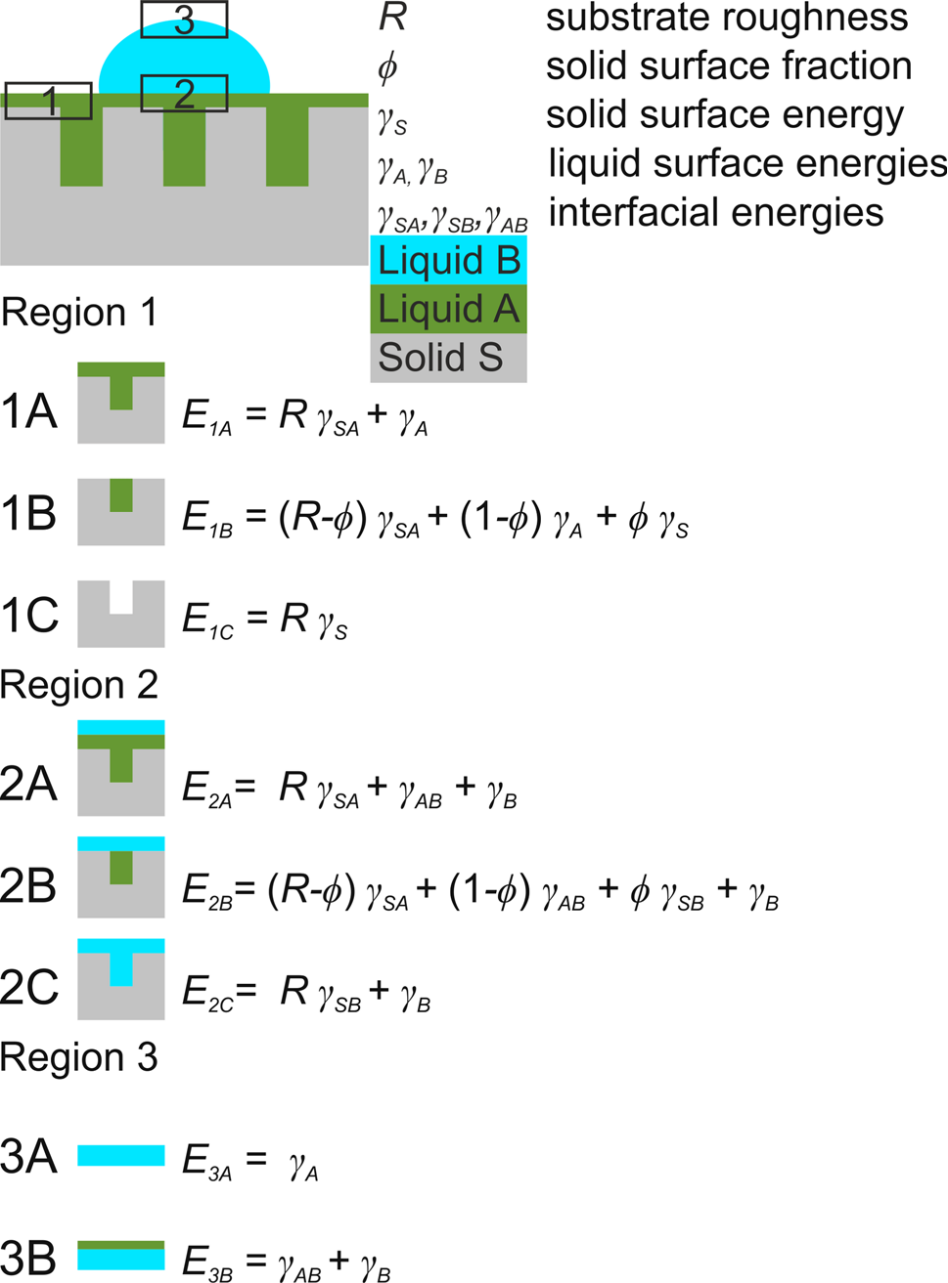
Interface energies *E*_(1–3)(A–C)_ per unit area for different wetting states (*A*–*C*) in different regions (1–3) of a structured solid substrate* S* infused by a liquid* A* and wetted by an immiscible liquid* B*

A model originally proposed in Ref. [37] is used in this work to describe the different wetting states of a liquid-infused substrate in contact with a second probe liquid (Fig. 2). A rough solid substrate S with a surface roughness *R* (ratio of the total surface area and the projected surface area), a solid surface fraction *ϕ* (fraction of solid per unit projected surface area), and a surface energy of *γ*_S_ is infiltrated with a liquid A (surface energy *γ*_A_) and is exposed to a probe liquid B (surface energy *γ*_B_) immiscible with A. The interface energies S/A, S/B, and A/B are denoted as *γ*_SA_, *γ*_SB_, and *γ*_AB_. Ideally, as shown in the top model in Fig. 2, liquid A completely fills the pores of the substrate and also completely covers the top solid surface fraction *ϕ*, and liquid B leaves this liquid-infused configuration undisturbed and forms a drop with a certain contact angle on the surface of liquid A. We will discuss in the following which conditions must be met to achieve this desired configuration and which other, unwanted wetting states can occur. For this purpose, we pick three representative regions in our model, denoted 1–3 in Fig. 2, and first calculate the total interface energies for all relevant wetting configurations in each of these regions. Region 1 is the three-phase range solid/liquid A/air that does not get in contact with liquid B. The three relevant wetting states are complete wetting of the outer surface *ϕ* and the inner surface *R* *−* *ϕ* (1A), a wetted inner surface and a dry outer surface (1B), and the completely dry substrate (1C). The corresponding interface energies per unit area for 1A–1C are listed in Fig. 2. Region 2 is located where the substrate infused with liquid A is covered by the second liquid B. The three relevant wetting states 2A–2C for this region are obtained simply by substituting air as the ambient medium in region 2 with liquid B. The corresponding expressions for the interface energies of states 2A–2C (Fig. 2) are therefore simply related to the energies of 1A–1C by the substitutions *γ*_B_ = 0, *γ*_AB_ = *γ*_A_, and *γ*_SB_ = *γ*_S_. Region 3 represents the outermost liquid/air interface of liquid B and can exist in one of two states: either as a liquid B/air interface (state 3A) or as liquid B/liquid A/air interface (state 3B). Thus, the corresponding expressions for the interface energies in region 3 contain only liquid surface energies and are independent of the substrate.

To find the conditions under which the different wetting states are thermodynamically stable, their interface energies are compared and the minimum energy states are determined as a function of some experimentally accessible parameters. For region 1, this gives the following set of three inequalities, which can be transformed into simple expressions for cos *θ*_A_ = $\frac{{\gamma_{\text{S}} - \gamma_{\text{SA}} }}{{\gamma_{\text{A}} }}$, where *θ*_Α_ is the contact angle of liquid A on the flat solid substrate S:

1 $$E_{{ 1 {\text{B}}}} < E_{{ 1 {\text{A}}}} \Leftrightarrow \frac{{\gamma_{\text{S}} - \gamma_{\text{SA}} }}{{\gamma_{\text{A}} }} < { 1,}$$

2 $$E_{{ 1 {\text{C}}}} < E_{{ 1 {\text{A}}}} \Leftrightarrow \frac{{\gamma_{\text{S}} - \gamma_{\text{SA}} }}{{\gamma_{\text{A}} }} < \frac{1}{R},$$

3 $$E_{{ 1 {\text{C}}}} < E_{{ 1 {\text{B}}}} \Leftrightarrow \frac{{\gamma_{\text{S}} - \gamma_{\text{SA}} }}{{\gamma_{\text{A}} }} < \frac{(1 - \varPhi )}{(R - \varPhi )}.$$

Exactly the same treatment of the interface energies for region 2 gives:

4 $$E_{{ 2 {\text{B}}}} < E_{{ 2 {\text{A}}}} \Leftrightarrow \frac{{\gamma_{\text{SB}} - \gamma_{\text{SA}} }}{{\gamma_{\text{AB}} }} < { 1,}$$

5 $$E_{{ 2 {\text{C}}}} < E_{{ 2 {\text{A}}}} \Leftrightarrow \frac{{\gamma_{\text{SB}} - \gamma_{\text{SA}} }}{{\gamma_{\text{AB}} }} < \frac{1}{R},$$

6 $$E_{{ 2 {\text{C}}}} < E_{{ 2 {\text{B}}}} \Leftrightarrow \frac{{\gamma_{\text{SB}} - \gamma_{\text{SA}} }}{{\gamma_{\text{AB}} }} < \frac{(1 - \varPhi )}{(R - \varPhi )} .$$

The independent parameter for the relative interface energies is now $\frac{{\gamma_{\text{SB}} - \gamma_{\text{SA}} }}{{\gamma_{\text{AB}} }}$ = cos *θ*_Α(Β)_, where *θ*_Α(Β)_ is the contact angle of liquid A on the flat solid substrate immersed in liquid B as the ambient medium. Somewhat simpler is the corresponding analysis of region 3:

7 $$E_{{ 3 {\text{A}}}} < E_{{ 3 {\text{B}}}} \Leftrightarrow \frac{{\gamma_{\text{B}} - \gamma_{\text{A}} }}{{\gamma_{\text{AB}} }} < { 1} .$$

**Fig. 3:**
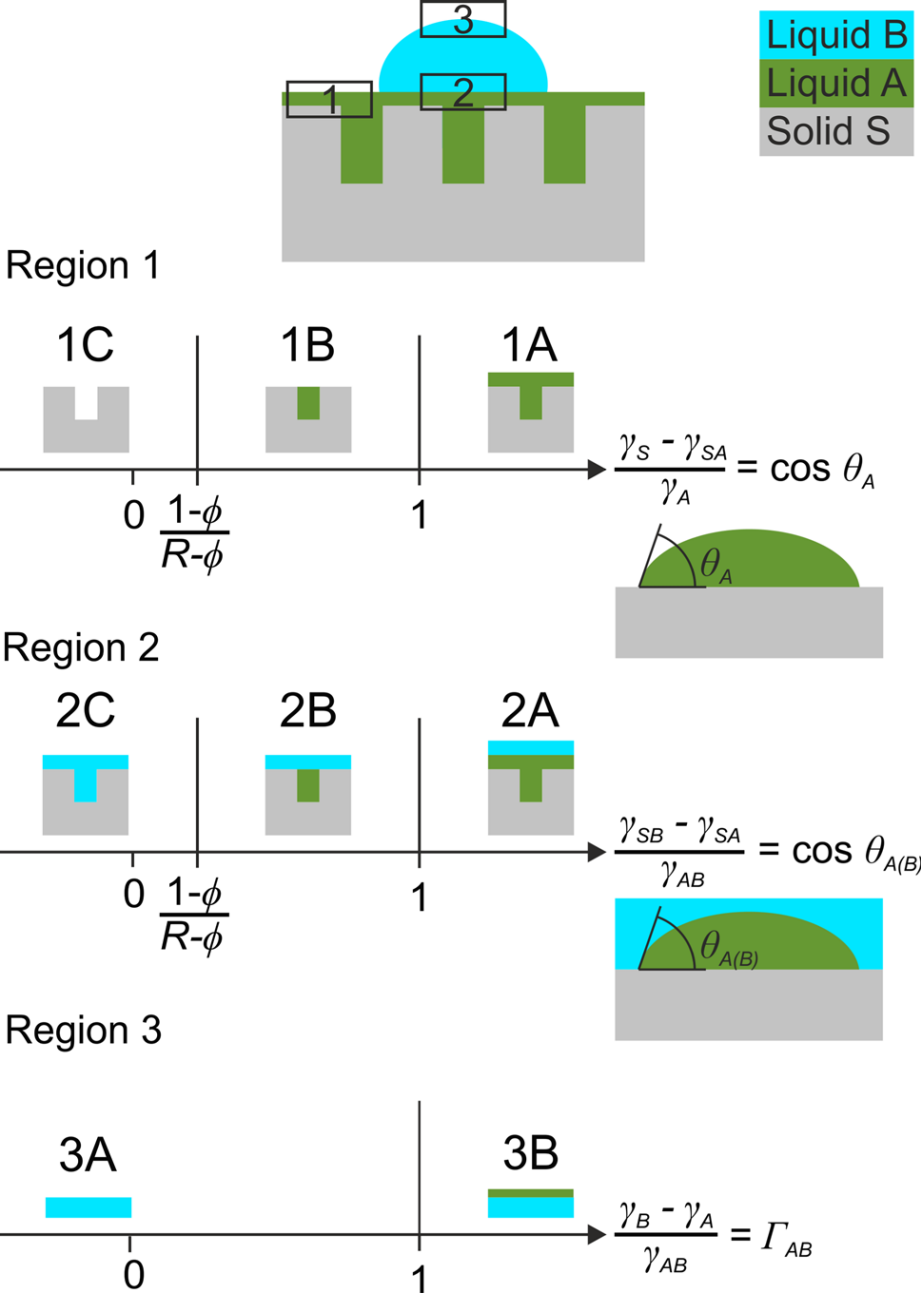
Stability ranges of different wetting states (*A*–*C*) in different regions (1–3) of a rough solid substrate* S* infused by a liquid* A* and wetted by an immiscible liquid* B*

The relative interface energies can now be plotted as a function of cos *θ*_Α_ for region 1, as a function of cos *θ*_Α(Β)_ for region 2, and as a function of $\frac{{\gamma_{\text{B}} - \gamma_{\text{A}} }}{{\gamma_{\text{AB}} }}$ = *Γ*_AB_ for region 3 to give energy diagrams of the relative stabilities of the different wetting states (Supplementary Material, Fig. S1). A simplified version of this energy level diagrams is shown in Fig. 3, where only the lowest energy states of Fig. S1 are considered. Thus, the wetting behavior of liquids on a liquid-infused substrate can be described by three easily accessible experimental parameters cos *θ*_Α_, cos *θ*_Α(Β)_, and *Γ*_AB_.

### Wetting state classifications

A liquid-infused substrate to be used as a liquid-repellent surface should fulfill the following requirements:

- The lubricating liquid A should completely fill the pores of the substrate and also cover the outer substrate surface (wetting state 1A in Fig. 3). The outer substrate surface is then a pure liquid with small contact angle hysteresis and without any solid protrusions where drops of the probe liquid B might get stuck.
- The probe liquid B should just float on top of liquid A and not displace liquid A and come in contact with the substrate at all (wetting state 2A in Fig. 3). A pure liquid/liquid interface and a single liquid/liquid/air contact line guarantee minimal interaction and maximal mobility of liquid B on the surface.
- Liquids of low surface tension tend to engulf (cloak) drops of other liquids, resulting in the wetting state 3B in Fig. 3. Cloaking must be avoided, because it not only reduces the mobility of liquid B on the surface, but also removes liquid A from the substrate, eventually exposing the blank solid substrate to liquid B concomitant with a loss of its liquid-repellent properties.

Using the notation of Fig. 3, the desired wetting state of a liquid-repellent, liquid-infused substrate is therefore the state (1A, 2A, 3A). The corresponding parameters are cos *θ*_A_ = 1 (*θ*_A_ = 0°), cos *θ*_A(B)_ = 1 (*θ*_A(B)_ = 0°), and *Γ*_AB_ < 1. We will discuss in the following if these requirements are practically achievable. Table 1 gives an overview of literature data on previously studied systems, where the parameters cos *θ*_A_, cos *θ*_A(B)_, and *Γ*_AB_ could be calculated from published data of contact angles and surface tensions. The systems are listed in Table 1 in the order of decreasing *Γ*_AB_ values. Apparently, the ideal wetting state 1A, 2A, 3A is not reached in any of them. The upper half of them have *Γ*_AB_ values ≥1 and suffer from the detrimental effects of cloaking (wetting state 3B). This includes many of the well-studied nonpolar SLIPS based on fluorinated substrate surfaces and polyfluorinated liquids like Krytox or FC70 as lubricating liquids [39]. The other groups in the lower part of Table 1 are polar SLIPS, which are characterized by a polar substrate like native silicon and a polar-infiltrating liquid like water. Only a few studies are known today about such polar liquid-infused systems, even though the parameters in Table 1 look promising: Especially the last example of native silicon infiltrated by water in contact with alkane solvents should come close to the ideal wetting state (1A, 2A, 3A). A serious drawback, however, is the high volatility of water. A viable alternative should be a “water-like”, hydrophilic ionic liquid with high surface tension. Based on these considerations, 1-ethyl-3-methylimidazolium methylsulfate ([C_2_mim]MeSO_4_) (*γ* = 55 mN m^−1^) was chosen for this study.

**Table 1:** Wetting parameters cos *θ*_Α_, cos *θ*_Α(Β)_, and *Γ*_AB_ of different liquid-infused substrates with a lubricating liquid A and a probe liquid B, calculated from published data of contact angles *θ*, surface/interface tensions *γ*, and surface roughness *R*

Solid	Liquid A	Liquid B	cos *θ*_A_	cos *θ*_A(B)_	*Γ*_AB_	References
Si-FDTS^a^	Krytox	Hexadecane	0.74	1.00	1.18	[38]^d^
Si-OTS^b^	Silicone	Water	1.00	1.00	1.12	[37]
Si-OTS^b^	Krytox	Water	0.88	0.88	1.12	[39]
Si-FDTS^a^	Krytox	Glycerol	0.74	1.00	1.06	[38]^d^
Si-FDTS^a^	Krytox	Water	0.74	0.89	0.98	[38]^d^
Si-FDTS^a^	Krytox	Heptane	0.74	0.79	0.66	[38]^d^
Si-OTS^b^	Bmim^c^	Water	0.49	0.98	0.62	[37]
Si-SiO_2_	Bmim^c^	Water	0.99	−0.68	0.62	[37]
Si-SiO_2_	Water	Hexane	0.97	1.00	−1.06	[18]^d^

^a^Silicon coated with perfluorodecyltrichlorosilane

^b^Silicon coated with octadecyltrichlorosilane

^c^1-Butyl-3-methylimidazolium bis(trifluoro-methyl-sulfonyl) imide

^d^cos *θ*_A(B)_ was calculated from the published Δ*E*_1_ values using the relationship Δ*E*_1_ = *γ*_AB_ (*R* cos *θ*_A(B)_ − 1)

### Wetting properties of [C_2_mim]MeSO_4_ on native and functionalized flat silicon

**Fig. 4:**
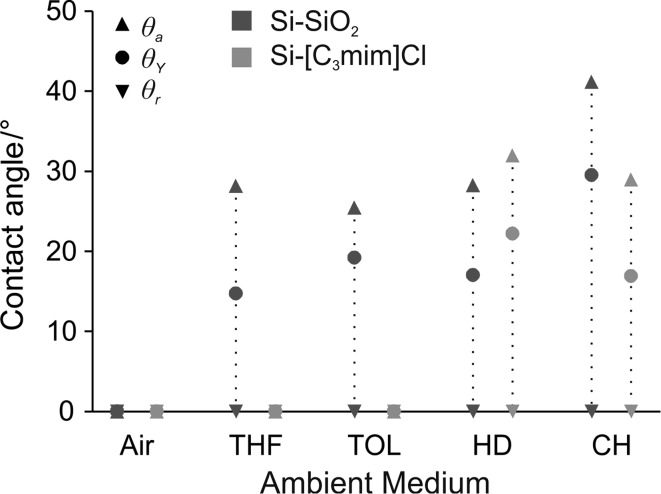
Static contact angles (*θ*_Y_) and dynamic contact angles (*θ*_a_, *θ*_r_) of the ionic liquid [C_2_mim]MeSO_4_ on native silicon (Si–SiO_2_) and on functionalized silicon (Si-[C_3_mim]Cl) in different ambient media (*THF* tetrahydrofuran, *TOL* toluene, *HD* hexadecane, *CH* cyclohexane)

To evaluate the suitability of different surface terminations of the silicon substrate for the infiltration with ionic liquid and the liquid-repellent properties of the resulting SLIPS for different probe liquids, the contact angles of [C_2_mim]MeSO_4_ were measured on uncoated, flat silicon wafers covered with a native oxide layer (Si–SiO_2_) and on wafers coated with a monolayer of 1-methyl-3-(3-trimethoxysilylpropyl)imidazolium chloride (Si-[C_3_mim]Cl) in air and probe liquid as ambient medium (Fig. 4). In air as the ambient medium, [C_2_mim]MeSO_4_ completely wets both the native silicon and the Si-[C_3_mim]Cl-functionalized flat silicon surface (*θ*_Y_ = 0°). Thus, cos *θ*_A_ = 1 and a rough silicon substrate with either a native oxide or a Si-[C_3_mim]Cl surface layer should be completely wetted by [C_2_mim]MeSO_4_ (wetting state 1A). Stable SLIPS can therefore be prepared with both substrates. In a liquid environment, however, only the Si-[C_3_mim]Cl functionalized surface remains fully wetted by the ionic liquid in THF and toluene as ambient solvents (*θ*_Y_ = 0°), resulting in cos *θ*_A(B)_ = 1 in these solvents and the desired wetting state 2A, where the probe liquid does not come in contact with the substrate at all. In all other systems included in Fig. 4, the static contact angles of [C_2_mim]MeSO_4_ lie between 10° and 30°, such that cos *θ*_A(B)_ < 1 and the probe liquid is predicted to partially replace the ionic liquid and come in direct contact with the solid substrate (wetting state 2B). A comparison of the IL contact angles in Fig. 4 between the uncoated, native substrate and the Si-[C_3_mim]Cl-coated substrate reveals that the contact angles are generally lower for the coated substrates. Thus, an Si-[C_3_mim]Cl monolayer increases the “IL-philic” properties of the surface and favors in binary liquid systems the wetting by the ionic liquid. Table 2 lists the parameters cos *θ*_A_, cos *θ*_A(B)_, and *Γ*_AB_ for all systems under study here. The *Γ*_AB_ values were calculated from measured surface and interface tensions, which are listed in Table 3. A comparison with the literature data in Table 1 shows that the parameters in Table 2 are much closer to the desired configuration cos *θ*_A_ = 1, cos *θ*_A(B)_ = 1, *Γ*_AB_ < 1.

**Table 2:** Wetting parameters cos *θ*_Α_, cos *θ*_Α(Β)_, and *Γ*_AB_ of nanoporous silicon infused with [C_2_mim]MeSO_4_ (liquid A) with respect to different probe liquids (liquid B)

Solid	Liquid A	Liquid B	cos *θ*_A_	cos *θ*_A(B)_	*Γ*_AB_
Si–SiO_2_	[C_2_mim]MeSO_4_	Tetrahydrofuran	1.00	0.97	−4.4
Si–SiO_2_	[C_2_mim]MeSO_4_	Toluene	1.00	0.94	−2.6
Si–SiO_2_	[C_2_mim]MeSO_4_	Hexadecane	1.00	0.86	−1.3
Si–SiO_2_	[C_2_mim]MeSO_4_	Cyclohexane	1.00	0.97	−1.6
Si[C_3_mim]Cl	[C_2_mim]MeSO_4_	Tetrahydrofuran	1.00	1.00	−4.4
Si-[C_3_mim]Cl	[C_2_mim]MeSO_4_	Toluene	1.00	1.00	−2.6
Si-[C_3_mim]Cl	[C_2_mim]MeSO_4_	Hexadecane	1.00	0.93	−1.3
Si-[C_3_mim]Cl	[C_2_mim]MeSO_4_	Cyclohexane	1.00	0.96	−1.6

The solid surface composition was either a native oxide (Si–SiO_2_) or a monolayer of Si-[C_3_mim]Cl

### Impregnation of nanoporous silicon with [C_2_mim]MeSO_4_

**Fig. 5:**
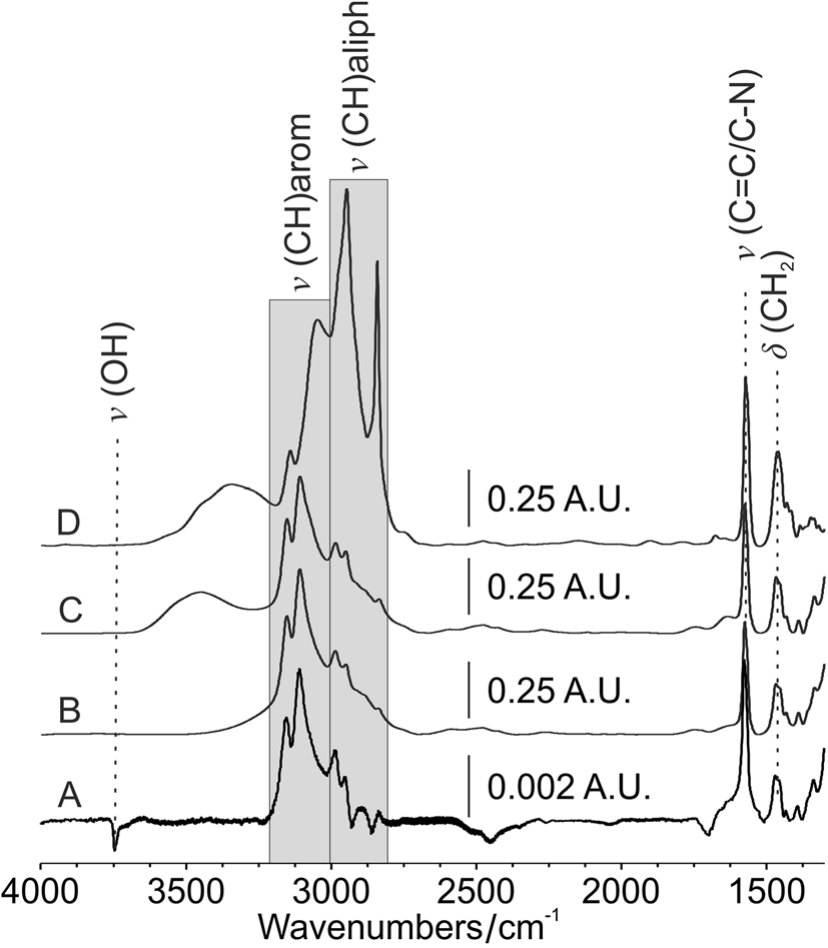
IR spectra of *a* nanoporous silicon coated with a monolayer of Si-[C_3_mim]Cl and *b* infused with the ionic liquid [C_2_mim]MeSO_4_. *c* Reference spectrum of neat [C_2_mim]MeSO_4_. *d* Reference spectrum of neat [(CH_3_O)_3_Si-[C_3_mim]Cl. Spectra *a* and *b* were referenced against uncoated nanoporous silicon, spectra *c* and *d* against the empty beam

A few drops of [C_2_mim]MeSO_4_ were deposited on nanoporous silicon substrates with either native oxide or Si-[C_3_mim]Cl surface termination. In both cases, the ionic liquid is sucked into the substrate pores and the excess liquid spreads evenly across the surface, in agreement with the behavior predicted by the wetting parameter cos *θ*_A_ = 1. Due to the extremely low volatility of the ionic liquid, a quasi-equilibrium state is reached when the excess liquid drains by gravity from the tilted substrate [27], leaving behind the impregnated, porous substrate with a liquid layer of a few micrometers thickness on top. Additionally, it was important here to saturate the infused ionic liquid with the particular probe liquid, because it has been shown that even minute quantities of dissolved cosolvents can change the surface tension and the wetting properties dramatically [39]. This was achieved here by immersing the substrate after IL impregnation vertically in the probe liquid. The whole process of coating and impregnation of the nanoporous substrates was monitored with FT-IR spectroscopy. Figure 5 shows IR spectra of the Si-[C_3_mim]Cl-coated substrates before impregnation (Fig. 5a) and after impregnation with [C_2_mim]MeSO_4_ and equilibration in toluene (Fig. 5b). For comparison, spectra of the coating precursor [(CH_3_O)_3_Si-[C_3_mim]Cl (Fig. 5d) and the neat ionic liquid [C_2_mim]MeSO_4_ (Fig. 5c) are also included. The low-frequency region of these spectra is essentially identical, showing the highly characteristic absorption around 1577 cm^−1^ due to the C–C/C–N ring stretching vibration of the imidazolium ring and the δ(CH_2_) deformation mode of the aliphatic hydrocarbon groups at about 1470 cm^−1^ [40, 41]. The CH stretching region shows absorptions of the aliphatic CH groups below 3000 cm^−1^ and the aromatic CH vibrations above 3000 cm^−1^ [41]. In addition, the Si-[C_3_mim]Cl monolayer spectrum shows a highly characteristic negative peak at 3747 cm^−1^, which indicates the removal of the free surface OH groups through covalent bond formation in the monolayer sample in comparison to the uncoated native silicon Ref. [42]. The disappearance of the free OH absorption in the monolayer-coated samples also indicates a complete and homogeneous coverage of the outer and inner substrate surface with Si-[C_3_mim]Cl. The CH stretching peaks in the monolayer spectrum are slightly different from the bulk reference spectrum (Fig. 5d) due to the overlap of additional methoxy group absorptions in the bulk spectrum. The ionic liquid spectra of the infused substrate saturated with toluene (Fig. 5b) and saturated with cyclohexane (not shown) are essentially identical to the reference spectrum of pure [C_2_mim]MeSO_4_ (Fig. 5c) and show no absorptions of dissolved cosolvent. Hexadecane and tetrahydrofuran, on the other hand, show strong solvent absorptions in the saturated ionic liquid layer, which are due to the formation of an insoluble, slowly evaporating thin film of cosolvent at the ionic liquid/air interface, which is formed upon removal of the IL infused substrate from the IL/solvent mixture.

### Liquid repellency of [C_2_mim]MeSO_4_-infused silicon

**Fig. 6:**
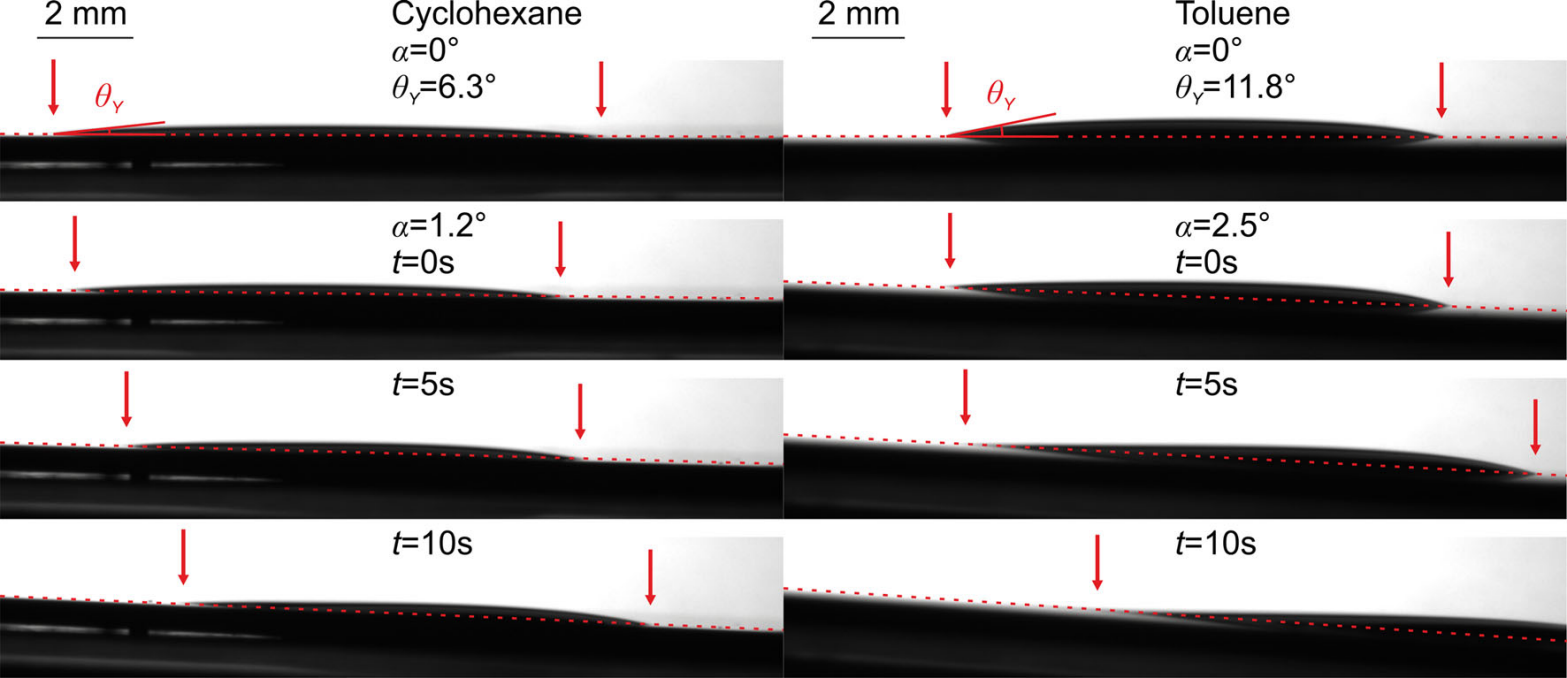
Droplet images of cyclohexane and toluene on a nanoporous Si–SiO_2_ substrate infused with [C_2_mim]MeSO_4_. The *upper row* shows the static drops on the horizontal substrate with the Young angles *θ*_Y_; the *lower rows* show a time sequence of the sliding drops beginning at the roll-off angle *α*. Drop dimensions are marked by *red arrows* (color figure online)

Nanoporous silicon substrates infused with [C_2_mim]MeSO_4_ and saturated with different probe liquids as described above were tested for their liquid repellency by depositing a drop of the probe liquid on the substrate surface and measuring first its static contact angle and then, by slowly tilting the substrate, the roll-off angle and the dynamic (advancing, receding) angles. Figure 6 shows images of a static and a moving drop (approximately, 20 mm^3^) of cyclohexane on the IL-infused substrate. Due to the high surface tension of [C_2_mim]MeSO_4_ in comparison to cyclohexane, the static contact angle is very small (*θ*_Y_ = 6.4° ± 2.9°) such that the drop is very flat and hardly visible in Fig. 6. Despite its large contact area with the IL-infused substrate, it is highly mobile and rolls off at a tilt angle of *α* = 1°. Advancing and receding angles were measured as *θ*_a_ = 8.7° ± 4.0° and *θ*_r_ = 4.0° ± 1.6°. Thus, Δ*θ* ≈ 4.7° and *α* = 1°, which are among the best liquid-repellent properties reported to date for low surface tension liquids. Very similar parameters were measured for toluene (Fig. 6; Δ*θ* ≈ 10.6° and *α* = 2.5°). Hexadecane and tetrahydrofuran, on the other hand, do not form drops and wet the ionic liquid surface completely (*θ*_Y_ = 0°). Notably, we did not observe any difference in the liquid-repellent properties between native silicon (Si–SiO_2_) and coated silicon substrates (Si-[C_3_mim]Cl), even though the cos *θ*_Α(B)_ parameters (Table 2) are different and predict partial replacement of the ionic liquid with both toluene and cyclohexane for the uncoated substrates. This should result in a noticeable increase of the roll-off angle *α* because of direct contact of the solvent with the solid surface. The fact that this was not observed in any of the systems investigated here suggests that the thermodynamic equilibrium state characterized by the static contact angles *θ*_Α(B)_ is not reached because of the kinetic barrier imposed by the receding angles. The ionic liquid can only be replaced by a solvent if the receding angle with the solvent as ambient medium *θ*_r, Α(B)_ > 0°. Since this is not the case in all systems studied here (Fig. 4), the ionic liquid never comes in contact with the solid and the ideal wetting state of a SLIPS substrate is maintained.

## Conclusions

Liquid-infused, solid substrates (so-called SLIPS) are a novel and promising approach for the fabrication of stable, liquid-repellent surfaces, but are based almost exclusively on the use of fluorinated compounds as lubricating liquids and as surface-coating materials. We have shown in this study that hydrophilic, fluorine-free ionic liquids are a comparatively cheap and non-toxic alternative as lubricating liquids that can be used for the impregnation of natural, uncoated oxide surfaces. Due to the higher surface tensions of hydrophilic ionic liquids, another inherent problem of SLIPS with fluorinated lubricants—the cloaking of the probe liquid—can be avoided. On the other hand, the miscibility of hydrophilic ionic liquids with other solvents is generally higher, and the currently known examples of liquid-repellent polar SLIPS are limited to a relatively small group of immiscible solvents. Further studies focusing on a combined optimization of wetting and solubility are required here, making use of the large variety of physicochemical properties that ionic liquids offer.

## Experimental

1-Methylimidazole (99% purity) and (3-chloropropyl)trimethoxysilane (>97% purity) were purchased from Sigma-Aldrich and used as received. The solvents toluene (Aldrich, >99.3%), hexadecane (Aldrich, 99%), cyclohexane (Aldrich, >99%), and tetrahydrofuran (Aldrich, >99%) were used as received.

Ionic liquids: 1-Methyl-3-(3-trimethoxysilylpropyl)imidazolium chloride ([(CH_3_O)_3_Si-C_3_mim]Cl) (**1**) was prepared according to a literature procedure [43]; analytical data were in accordance with literature [43]. 1-Ethyl-3-methylimidazolium methylsulfate ([C_2_mim]MeSO_4_) (**2**) was prepared by adding 12.60 g methyl sulfate (100 mmol) dropwise to a solution of 9.60 g freshly distilled 1-ethylimidazole (100 mmol) in 100 cm^3^ dichloromethane under cooling at in an NaCl/ice bath. The solution was stirred at room temperature for 6 h and the solvent was removed under reduced pressure. The obtained liquid was dried in vacuo (1 × 10^−2^ mbar) with stirring at 50 °C for 24 h; 22.20 g (>99%) of a colorless liquid was yielded; analytical data were in accordance with literature [44].

**Fig. 7:**
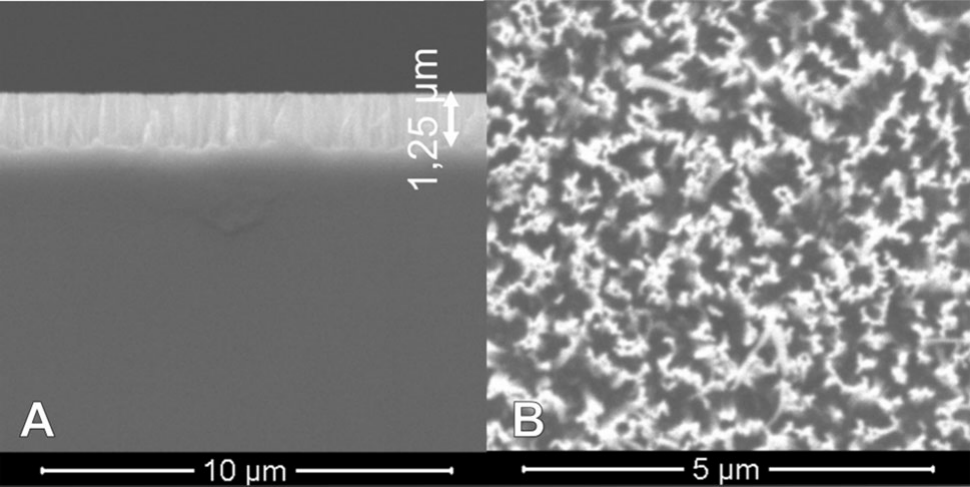
Cross-sectional (**a**) and top view (**b**) SEM images of a nanoporous silicon substrate prepared by silver-catalyzed etching in HF/H_2_O_2_ solution

Silicon wafers ((100)-oriented, p-doped, 10–20 Ω cm, 500–550 µm thickness, double-sided polished) were purchased from MEMC Electronic Materials. They were cut into 18 × 25 mm rectangular pieces and cleaned by sonication in toluene and UV-ozone treatment in a commercial cleaning chamber (Boekel Industries, UVClean). For the preparation of liquid-infused substrates, a nanoporous surface layer was created by silver-catalyzed electroless chemical etching [45] using aqueous H_2_O_2_/HF solutions as the etchant. The cleaned Si substrates were immersed in 20 cm^3^ aqueous solution of 17.0 mg silver nitrate (5 mmol/dm^3^) and 4.5 cm^3^ hydrofluoric acid (48%, 6.2 mmol/dm^3^) for 1 min to deposit a layer of Ag nanoparticles to act as the catalyst in the etching process. After rinsing with water, the substrates were etched for 10 min in 20 cm^3^ of an aqueous solution of 4.5 cm^3^ HF (48%, 6.2 mmol/dm^3^) and 100 mm^3^ of 36% H_2_O_2_ (0.06 mol/dm^3^). The silver catalyst was finally dissolved by immersion in nitric acid (35%) for 5 min and the samples were rinsed with water, immersed in piranha solution (4:1 H_2_SO_4_ 96%/H_2_O_2_ 36%), rinsed again with water and acetone and blow-dried with argon. This procedure yields a nanoporous surface layer of about 1 μm thickness consisting of vertical, uniformly oriented nanowires of about 20–50 nm diameter separated by vertical pores of similar dimensions (Fig. 7).

### Surface modification

The surface composition of both flat and nanoporous Si substrates in this study was either a surface oxide layer of about 1–2 nm thickness resulting from the UV-ozone treatment during the cleaning process or an adsorbed monolayer of 1-methyl-3-(3-trimethoxysilylpropyl)imidazolium chloride (**1**). The adsorption was carried out by immersing the cleaned substrates (flat or nanoporous) in a solution of 28.1 mg (10^−4^ mol) of **1** in 10 cm^3^ toluene for 2 days. The samples were rinsed with toluene and immersed in 10 cm^3^ ethanol for 3 days to remove any physisorbed material. The samples were finally rinsed with ethanol and blow dried with argon. The thickness of the resulting films on flat silicon wafers were measured by ellipsometry and gave values of 4.4 ± 0.6 Å, in agreement with the formation of a monolayer film. On nanoporous substrates, FT-IR was used to check the completeness of monolayer adsorption.

### Surface infiltration

Freshly etched nanoporous silicon substrates were wetted on both sides with the ionic liquid [C_2_mim]MeSO_4_ and the liquid was allowed to be sucked into the nanopores by capillary forces. To remove excess liquid and equilibrate the infused ionic liquid with a particular probe liquid (toluene, hexadecane, cyclohexane, tetrahydrofuran), the substrates were immersed vertically for 7 days in a two-phase mixture of 1 cm^3^ of ionic liquid (covering just the very bottom of the immersed substrates) and 10 cm^3^ probe liquid. The samples were removed from the liquid mixture, the excess ionic liquid at the bottom of the substrate was carefully dabbed off and the substrates were immediately transferred into the sample chamber for contact angle measurements.

### NMR spectroscopy

^1^H NMR and ^13^C NMR spectra were recorded on a Bruker AVANCE 250 spectrometer. The spectra were referenced internally to residual protio-solvent and solvent resonances, respectively, and were reported relative to tetramethylsilane (*δ* = 0 ppm).

### IR spectroscopy

Infrared spectra were measured on a Bruker Vertex 80 FT-IR spectrometer using either a DTGS detector or a narrow band MCT detector. Between 16 and 256 scans at 4 cm^−1^ resolution were recorded for sample and reference, respectively. Dry and liquid-infused porous silicon substrates were measured in transmission at an incidence angle of 40° to suppress residual interference fringes. Spectra of monolayer-coated porous silicon substrates were referenced against spectra of the uncoated substrate. Spectra of liquid reference compounds were measured as thin films between sodium chloride plates.

### Ellipsometry

Ellipsometric measurements of coated and uncoated flat silicon samples were carried out using a Sentech SE 500adv single wavelength ellipsometer equipped with a He–Ne laser and a rotating analyzer. The measured ellipsometric angles were converted into film thickness using the commercial instrument software based on the McCrackin algorithm. Optical constants of *n* = 3.864 and *k* = 0.02 for Si, *n* = 1.465 and *k* = 0 for SiO_2_, and *n* = 1.50 and *k* = 0 for the organic coating layer were used. Measurements were carried out at four different spots across the sample surface and the variations were always below 1 Å.

### Scanning electron microscopy (SEM)

SEM images were recorded on a FEI Quanta 200 MK2 electron microscope equipped with an Everhardt–Thornley detector. Working distances between 8 and 11 mm and an electron beam voltage of 10 keV were used. For cross-sectional measurements, the samples were freshly cleaved along a crystallographic axis with a diamond tip and were mounted with the cross section perpendicular to the electron beam.

### Contact angle and surface/interface tension measurements

Contact angles and surface/interface tensions were measured on a Krüss DSA 30 contact angle goniometer equipped with a CCD video camera (resolution 780 × 582 pixel, speed 60 fps) and an inclinable sample stage with software-controlled tilting speed. A custom-built sample housing was used to carry out the contact angle and surface tension measurements in an atmosphere saturated with the probe liquid to minimize errors due to evaporation effects. Static contact angles were measured by putting a drop of probe liquid onto the sample surface, recording the drop image, and determining the contact angle using the tangent method (*θ* < 10°) or the Young–Laplace method (*θ* > 10°) of the instrument software. Advancing and receding angles were measured using the tilted plate method [46] by tilting the sample stage at a rate of 10°/min and continuously recording the image until the drop rolled off the surface. From the last image of the adhering drop, the advancing and receding angles were determined using the tangent method of the Krüss Advance software. Surface tensions were measured with the pendant drop method and a Young–Laplace drop shape analysis of the hanging drop in air as the surrounding medium. Interface tensions of the ionic liquid [C_2_mim]MeSO_4_ in contact with different immiscible or partly miscible solvents were measured also by drop shape analysis of a pendant IL droplet submersed in the different solvents. Table 3 lists the surface tensions and interface tensions of all liquids and liquid mixtures employed in this study.

**Table 3:** Surface tensions of different liquids and saturated liquid mixtures (A(B) → surface tension of liquid A saturated with B) and interface tensions of mutually saturated liquids

Liquid	Surface tension/10^−3^ N m^−1^	Interface tension/10^−3^ N m^−1^
[C_2_mim]MeSO_4_ ()	55.2 ± 0.5	
[C_2_mim]MeSO_4_—TOL		12.2 ± 0.3
TOL ([C_2_mim]MeSO_4_)	25.0 ± 0.1	
[C_2_mim]MeSO_4_ (TOL)	56.6 ± 0.4	
[C_2_mim]MeSO_4_—HD		20.5 ± 0.4
HD ([C_2_mim]MeSO_4_)	22.9 ± 0.4	
[C_2_mim]MeSO_4_ (HD)	51.1 ± 0.6	
[C_2_mim]MeSO_4_—CH		18.2 ± 0.4
CH ([C_2_mim]MeSO_4_)	25.3 ± 0.1	
[C_2_mim]MeSO_4_ (CH)	53.4 ± 0.7	
[C_2_mim]MeSO_4_—THF		7.4 ± 0.2
THF ([C_2_mim]MeSO_4_)	23.3 ± 0.2	
[C_2_mim]MeSO_4_ (THF)	55.8 ± 0.6	

Data represent mean values of three independent measurements

## Electronic supplementary material

Below is the link to the electronic supplementary material.

Supplementary material 1 (DOCX 5311 kb)
