# Supplementary material for: Fluorine-free, liquid-repellent surfaces made from ionic liquid-infused nanostructured silicon
Source: Monatsh Chem. 2016 Dec 18;148(1):167–77. doi: 10.1007/s00706-016-1888-2 (PMC5225213; doi:10.1007/s00706-016-1888-2)
Supplement: Supplementary file 1 — Supplementary material 1 (DOCX 5311 kb) [file 706_2016_1888_MOESM1_ESM.docx]

Supplementary Material

Fluorine-Free, Liquid-Repellant Surfaces made from Ionic-Liquid-Infused Nanostructured Silicon

**Roland Bittner^1^ ● Katharina Bica^1^ ● Helmuth Hoffmann^1^**

___

🖂 Helmuth Hoffmann

helmuth.hoffmann@tuwien.ac.at

^1^ Institute of Applied Synthetic Chemistry, Vienna University of Technology, Vienna, Austria





**Fig. S1** Relative interface energies and stability ranges of the different wetting states of a liquid-infused substrate. The wetting states and their interface energies are presented in Fig.2 of the original article.
